# One-Pot Facile Synthesis of a Cluster of ZnS Low-Dimensional Nanoparticles for High-Performance Supercapacitor Electrodes

**DOI:** 10.3390/mi15020251

**Published:** 2024-02-07

**Authors:** Sagar M. Mane, Komal S. Wagh, Aviraj M. Teli, Sonali A. Beknalkar, Jae Cheol Shin, Jaewoong Lee

**Affiliations:** 1 Department of Fiber System Engineering, Yeungnam University, 280 Dehak-Ro, Gyeongsan 38541, Gyeongbuk, Republic of Korea; 2 Independent Researcher, Gyeongsan 38544, Gyeongbuk, Republic of Korea; 3 Division of Electronics and Electrical Engineering, Dongguk University-Seoul, Seoul 04620, Republic of Korea

**Keywords:** solvothermal synthesis, ZnS, low-dimensional nanoparticles, high-performance supercapacitor, charge storage mechanism

## Abstract

To maximize the use of ZnS low-dimensional nanoparticles as high-performance supercapacitor electrodes, this work describes a simple one-pot synthesis method for producing a cluster of these particles. The ZnS nanoparticles fabricated in this work exhibit a cluster with unique low-dimensional (0D, 1D, and 2D) characteristics. Structural, morphological, and electrochemical investigations are all part of the thorough characterization of the produced materials. An X-ray diffraction pattern of clustered ZnS nanoparticles reflects the phase formation with highly stable cubic blende sphalerite polymorph. The confirmation of nanoparticle cluster formation featuring multiple low-dimensional nanostructures was achieved through field emission scanning electron microscopy (FE-SEM), while the internal structure was assessed using transmission electron microscopy (TEM). Systematically assessing the ZnS nanoparticles’ electrochemical performance reveals their prospective qualities as supercapacitor electrode materials. The electrode assembled with this material on Ni foam demonstrates elevated specific capacitance (areal capacitance) values, reaching 716.8 F.g⁻^1^ (2150.4 mF.cm^−2^) at a current density of 3 mA.cm⁻^2^. Moreover, it reflects 69.1% capacitance retention with a four times increase in current density, i.e., 495.5 F.g^−1^ (1486.56 mF.cm^−2^) capacitance was archived at 12 mA.cm^−2^ with 100% Coulombic efficiency. Furthermore, the electrode exhibits prolonged cycling capability with 77.7% capacitance retention, as evidenced by its charge–discharge measurements sustained over 15,000 cycles at a current density of 25 mA cm⁻^2^.

## 1. Introduction

To secure a sustainable future for humanity, it is imperative to address the escalating impacts of air pollution and global warming. This can be achieved by identifying and implementing ample, environmentally friendly, and renewable energy sources. These alternatives should offer viable options to fossil fuels, which are predominantly accountable for causing irreversible damage to the environment [1,2,3]. As a result, research focuses on the generation of energy through renewable sources, like solar and wind power. Additionally, notable emphasis is devoted to the conversion and storage of this energy through batteries, capacitors, and electrochemical supercapacitors, which has gained exponential interest in the past few decades [4]. Supercapacitors are considered emerging energy storage solutions, as they preserve richer features for this application. These characteristics encompass significantly high specific capacitance, rapid charge and discharge rates, prolonged lifespan, the ability to function in a wide temperature range, and more [5]. Supercapacitors have promising potential across new eco-friendly energy devices, information communication technology, and electric vehicles. However, their application in these technologically significant devices faces a challenge due to their low energy density [6,7]. To achieve enrichment in the energy density of supercapacitors, the three primary strategies need to be considered: (a) enhance system capacitance by refining the surface properties of electrode materials; (b) increase device voltage with proper electrodes and electrolytes; (c) fabricate hybrid systems/materials with which it is possible to achieve simultaneous elevation in capacitance and voltage [8]. The overall electrode materials have to be first taken into account while considering enhancement in the energy storage features of supercapacitors. In recent decades, transition metal sulfides (TMSs) have outperformed individual metal oxides in electrochemical performance. WS_2_, SnS_2_, Sb_2_S_3_, FeS_2_, ZnS, MnS, Co_3_S_4_, and Ni_3_S_2_ are the few promising TMSs that have been studied as electrode materials for energy storage applications [9,10,11,12]. Their intriguing properties, such as high theoretical capacitance and electronic conductivity, with other benefits like cost-effectiveness and abundance in nature, make them promising candidates to meet the demanding energy storage requirements of supercapacitors. These materials are regarded as dependable and auspicious substitutes for electrodes in the upcoming generation of pseudocapacitors [13,14]. Zinc sulfide (ZnS) stands out as a versatile semiconductor with a wide band gap, garnering significant attention in various fields such as nanogenerators, sensors, energy storage devices, light-emitting diodes, photodetectors, transistors, photocatalysis, infrared windows, bio-devices, and piezotronics [15,16,17]. In contrast to other transition metal sulfides, ZnS-based electrodes are known to exhibit charge storage capacity based on the redox conversion mechanism as well as the alloying mechanism [10]. This heightened interest is attributed to its commendable electrical conductivity, favorable electronic properties, stability, and diverse range of morphological characteristics [18]. Recent studies have recognized that the intrinsic characteristics (physical and chemical) of electrode materials play a crucial role in influencing pseudo-capacitive features. Additionally, the nanostructuring or deliberate engineering of these materials into low-dimensional nanostructures can greatly improve both electrical and ionic conductivity and also help to resolve the issues related to volume expansion [19,20,21,22]. Low-dimensional (0D, 1D, and 2D) architectures of various materials such as nanoparticles have swiftly emerged as top choices for supercapacitor electrodes. The predominant physical features such as a high degree of flexibility and stability, and exceptional electrical characteristics such as outstanding conductivity make them favorable for electrode materials. Therefore, supercapacitor devices based on these materials have gained interest in tremendous high-tech applications such as wearable electronics and microrobots [23]. Furthermore, due to their compactness, the low-dimensional materials are technologically significant for miniatured devices, stretchable energy systems, and nanoelectromechanical applications [24,25]. It has been observed that there are multiple synthetic methods available for the realistic design of low-dimensional nanostructures across a range of materials. Template-directed synthesis, hydrothermal/solvothermal approach, thermal evaporation, solution-phase methods, melt-blown techniques, sol-gel techniques, electrospinning, self-assembly processes, and electrodeposition are a few of these methods [26,27]. The hydrothermal/solvothermal technique is regarded as a promising pathway for the fabrication of various low-dimensional nanostructures of different materials compared to other synthetic approaches. This method offers several benefits; as the reaction is being held at a low temperature, this method will help reduce the crystal defects and thermal stresses, and due to the sealed vessel reaction, there are minimal pollution concerns and safety-related issues. Additionally, this technique of synthesis is characterized by its simplicity, cost-effectiveness, and ability to control the size and shape of the synthesized materials [28].

This research aims to illuminate the potential of the multiple low-dimensional ZnS nanoparticles as effective materials for supercapacitor electrodes. Through this study, we introduce a novel one-pot solvothermal synthetic strategy for generating multiple low-dimensional nanoparticles of zinc sulfide (ZnS). It is noted that particles of multiple dimensions give rise to a synergistic effect; as a result, a fascinating electrochemical performance was achieved for this electrode material. The electrode of ZnS (ZnS@Ni foam) exhibits 716.8 F gm^−1^ (2150.4 mF cm^−2^) capacitance at 3 mA cm^−2^ and excellent stability of 77.7% after 15,000 cycles, measured at 25 mA cm^−2^. Examining the charge storage mechanism thoroughly affirms that the capacitive charge storage prevails in this material.

## 2. Experimental Section

### 2.1. Materials

The required chemicals for the synthesis of ZnS, i.e., Zn(CH_3_COO)_2_·2H_2_O (zinc acetate dihydrate), C_2_H_5_NS (thioacetamide), and C_2_H_5_OH (ethanol) and for fabrication of its electrode on Ni-foam polyvinylidene fluoride (PVDF), and N-Methyl-2-pyrrolidone (NMP) were procured from Sigma-Aldrich and utilized in their original state.

### 2.2. Synthesis of Multiple Low-Dimensional ZnS Nanoparticles

The powdered sample of ZnS nanoparticles with multiple low-dimensional structures was fabricated through a solvothermal synthetic route. Initially, 1 mmol of Zn(CH_3_COO)_2_·2H_2_O was blended in a beaker containing 80 mL of solvent, comprising a 50:50 volume ratio of ethanol and distilled water. The solution mixed with Zn molecules underwent continuous stirring for a duration of 15 min and then 6 mmol C_2_H_5_NS added to it. Following sustained agitation for an additional 15 min, the solution was transferred to a Teflon vessel, which was further kept and sealed in a steel autoclave chamber. The complete reaction was achieved by subjecting this chamber to a temperature of 180 °C for 16 h in an oven. After the reaction period concluded, the ZnS precipitate underwent filtration and was then vacuum-dried overnight at 90 °C. This dried powder of ZnS was further assessed through various techniques and electrochemical tests were carried out after fabricating its electrode on Ni-foam.

### 2.3. Characterization Techniques

Phase formation of ZnS was confirmed by X-ray diffraction (DIATOME-Pananlytical, Malvern, UK). X-ray photoelectron spectroscopy (XPS; Versaprobe II, ULVAC-PHI Inc., Chigasaki, Kanagawa, Japan) was used to confirm the chemical states of Zn and S elements. A field emission scanning electron microscope (FE-SEM; S-4800, Hitachi, Ibaraki, Japan) and field emission transmission electron microscopy with EDS (FE–TEM; Tecnai G2 F20 S–TWIN, FEI, Hillsboro, OR, USA) were utilized to confirm multiple low-dimensional nanoparticles of ZnS and contained elements. Surface area and pore diameter were investigated through N_2_ adsorption–desorption using BET (Micromeritics; 3-flex analyzer, Norcross, GA, USA).

### 2.4. Electrode Fabrication and Electrochemical Measurements

A combination of ZnS nanoparticles, PVDF, and carbon black was prepared in a proportion of 80:10:10, resulting in the formulation of slurries. This mixture was applied onto a 3D nickel foam of 1 cm^2^ area that had been thoroughly cleaned through ultrasonic treatment with acetone, water, and ethanol. ZnS nanoparticle-coated Ni-foam underwent overnight drying at 80 °C and was used further as an electrode to investigate energy storage features. The weight of ZnS (active material) on Ni-foam was 3 mg, which was estimated through calculating Ni-foam weight before deposition and after drying. To investigate the energy storage performance of ZnS nanoparticles, electrochemical tests such as cyclic voltammetry, charge–discharge profile, and electrochemical impedance spectroscopy were performed. These evaluations were conducted in a 2 mol/L KOH aqueous solution and 3-electrode system (Pt wire-counter electrode, Ag/AgCl-reference electrode), using ZIVE SP5 (WonaTech; Seocho-gu, Seoul, Republic of Korea) potentiostat/galvanostat/impedance analyzer to determine the electrochemical characteristics of ZnS.

## 3. Results and Discussion

### 3.1. Structural and Surface Composition Analysis

**Figure 1:**
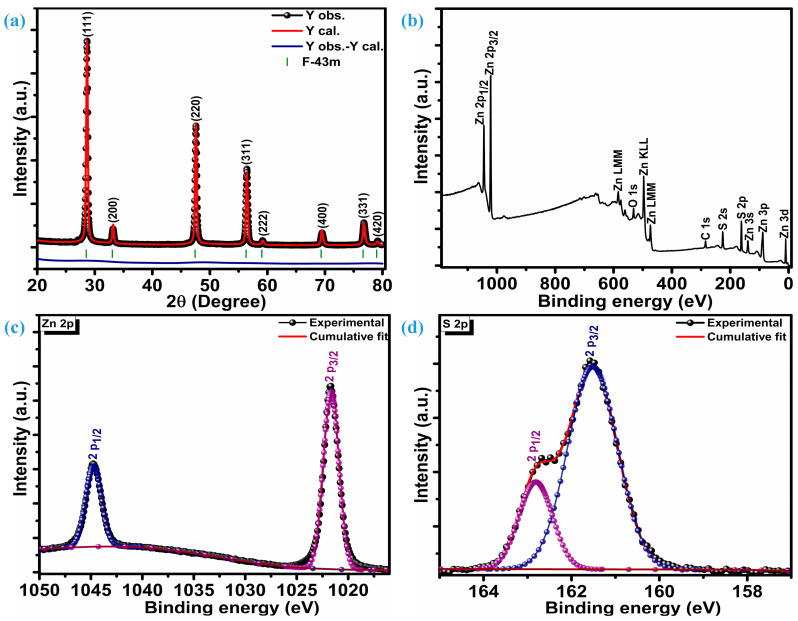
Structural and surface composition analysis, (**a**) diffraction patterns, (**b**) XPS survey spectra, (**c**) Zn 2p spectra, (**d**) S 2p spectra.

Figure 1a illustrates the crystalline reflections of multiple low-dimensional ZnS nanoparticles measured at room temperature. Along with three highly intense crystal planes, five other planes were observed when the ZnS sample was scanned at 2θ, ranging from 20 to 80°. The three intense planes were located at 28.7°, 47.6°, and 56.5° while other planes were situated at 33.1°, 59.2°, 69.6°, 76.8°, and 79.2° 2θ locations, respectively. Three highly intense sharp diffractions represent (111), (220), and (311) crystal planes, while other low peaks are assigned to (200), (222), (400), (331), and (420) planes, respectively. These diffraction peaks confirm ZnS crystallization into a highly stable sphalerite cubic blende phase with space group F-43m [10,29], which is, accordingly, JCPDF card number 01-086-8469. The distinct diffraction peaks devoid of any impurities indicate a high level of crystallinity and the exclusive presence of the ZnS material’s pure phase characteristics. To get insight into phase purity, XRD patterns of the ZnS were analyzed through Rietveld refinement of crystal structure analysis. Refinement indicates that the diffraction patterns of ZnS are well aligned with space group F-43m and space group number 216. The lattice parameters estimated through refinement are a = b = c = 5.41626 Å, and volume (a^3^) is 158.891 Å^3^. Refinement parameters R_wp_ = 5.25%, R_p_ = 3.74%, and χ^2^ = 2.30 confirm the refinement is successful. The determination of the average crystallite size (*D_A_*) involved the analysis of three particularly strong planes and the application of the Debye–Scherrer equation [30];

(1) $$D_{A}=\frac{0.9\lambda }{\beta \cos\theta }$$

The average crystallization size of ZnS nanoparticles remains at 24.6 nm.

X-ray photoelectron spectroscopy (XPS) was utilized to investigate the chemical composition, purity, and surface chemical states of multiple low-dimensional nanostructures of ZnS. Figure 1b displays survey spectra of the ZnS; XPS findings reveal distinct peaks corresponding to Zn, S, C, and O. Various oxygen species such as surface hydroxyl oxygen (OH), lattice oxygen (OL) in ZnO, and chemisorbed oxygen (OA) attributes to the O peak [31]. Additionally, a Gaussian fitting approach was employed to precisely fit the characteristic peaks of the Zn and S elements. Figure 1c represents high-resolution core-level spectra of the Zn 2p. Deconvolution confirms that the Zn 2p core-level spectrum has only two distinguished peaks, representing 2p_3/2_ and 2p_1/2_ levels of the Zn element. These two levels are centered at a binding energy of 1044.8 eV and 1021.7 eV, respectively, marking a spin–orbit splitting of 23.1 eV between them. This alignment of the spin-orbit splitting is consistent with that observed in Zn^2+^ [32]. The S 2p core-level spectrum deconvolution reveals two peaks positioned at 161.5 eV and 162.8 eV confirming the existence of the bivalent S^2-^ state with two levels 2p_3/2_ and 2p_1/2_, respectively. This is substantiated by the distinctive peak separation of 1.3 eV in between the centers of the two levels, i.e., 2p_3/2_ and 2p_1/2_, as seen in Figure 1d. The bonding between Zn and S (Zn-S) can be revealed through the peak centered at 161.5 eV [33]. X-ray photoelectron spectroscopy (XPS) unveils the atomic compositions of Zn and S as 57.75% and 42.25%, respectively. Therefore, it can be inferred that the formation of ZnS occurs in a ratio of 1.27:0.73 (Zn:S).

### 3.2. FE-SEM and TEM Analysis

**Figure 2:**
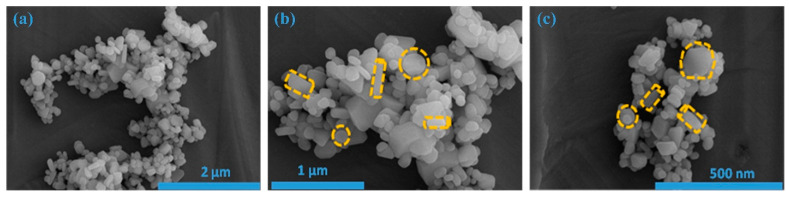
FE-SEM images of ZnS at different magnifications. (**a**) 2 μm; (**b**)1 μm; (**c**)500 nm.

Figure 2a–c illustrates field emission scanning electron microscopy (FE-SEM) images showcasing the as-prepared ZnS sample. FE-SEM images are taken at various magnifications, and these images unveil the emergence of easily distinguishable, low-dimensional nanostructures. To be more precise, when examining Figure 2a, one can observe a cluster of nanoparticles with various shapes/dimensions. This was additionally verified by examining the images at increased magnifications, as represented in Figure 2b,c.

**Figure 3:**
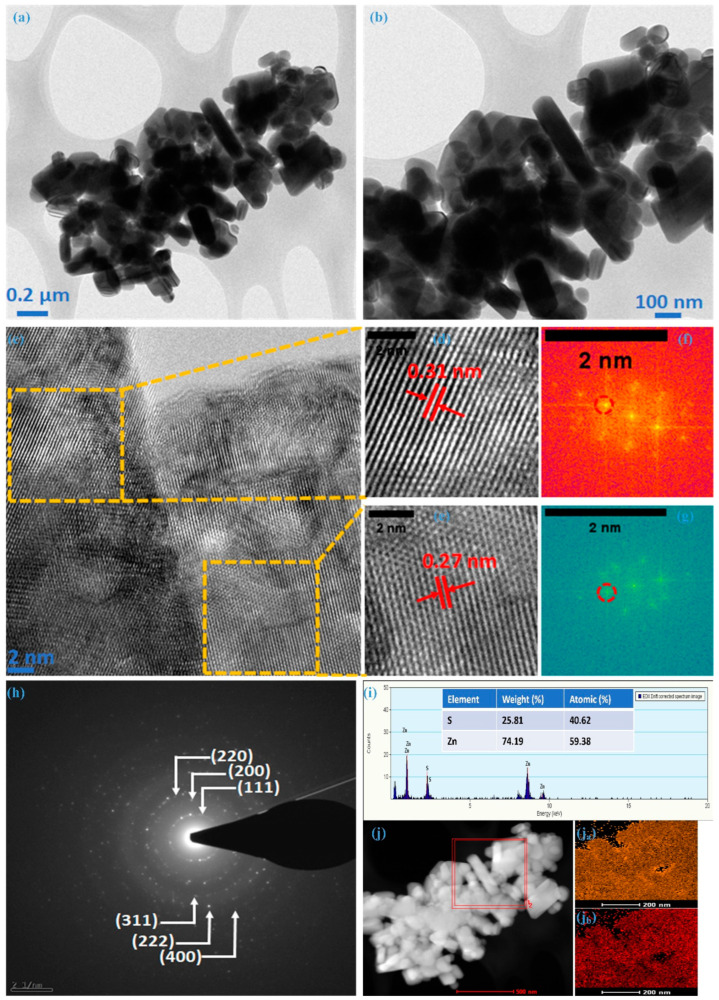
TEM and HR-TEM analysis of ZnS, (**a**,**b**) TEM images at different magnification, (**c**) HR-TEM image, (**d**) 0.31 nm fringe pattern, (**e**) 0.27 nm fringe pattern, (**f**) FFT for 0.31, (**g**) FFT for 0.27, (**h**) SAED for ZnS, (**i**) EDS, (**j**) HAADF, (**j_a_**) mapping of Zn, and (**j_b_**) mapping of S.

Transmission electron microscopy confirmed the internal morphological and structural evolution of the ZnS sample. TEM images at different magnifications are illustrated in Figure 3a,b, revealing the non-uniform nature of nanoparticles, exhibiting various dimensions; these images represent the silhouettes observed in the FE-SEM analysis. Figure 3c depicts a high-resolution transmission electron microscopy (HRTEM) image showcasing one such particle from the ZnS sample. The lattice fringes in this picture indicate that these multiple-dimensional particles are polycrystalline. Exemplifying a common instance of such particle, the zinc-blende structure was confirmed for ZnS with distinct fringes with an interplanar spacing (d-spacing) of 0.31 nm (Figure 3d and 0.27 nm (Figure 3e), respectively. These fringe patterns are associated with the most intense crystal plane (111) and its adjacent plane (200), respectively. This was further confirmed through spectral patterns (encircled) of the chosen region with different fringes using fast Fourier transform spectroscopy (FFT), as illustrated in Figure 3f,g. The selected area electron diffraction (SAED) pattern, as represented in Figure 3h for the ZnS sample synthesized in this work, exhibits distinct concentric rings corresponding to lattice planes (111), (200), (220), (311), and (222), aligning seamlessly with the cubic structural arrangement. This suggests that TEM and HR-TEM observations align closely with those made using FE-SEM and XRD. Analyzing synthesized nanoparticles through energy-dispersive X-ray spectroscopy (EDX) is a crucial method for determining the atomic percentages of the constituent elements. In Figure 3i, the presented image and data sheet derived from the EDX analysis provide valuable insights. The images distinctly reveal the presence of key elements, i.e., Zn and S without any additional peaks, indicating the formation of ZnS with a 1.31:0.69 (Zn:S) ratio. It suggests that the results obtained from the XPS analysis and EDS align well with the intended elemental composition of the material. Figure 3j reflects the elemental mapping using HAADF-STEM for Zn and S; as illustrated in Figure 3j_a_,j_b_, the homogeneous distribution of Zn and S atoms can be noted within the cluster of nanoparticles. Morphological analysis through FE-SEM and TEM verify the creation of ZnS, featuring nanoparticles with multiple dimensions. The predominant presence of multi-dimensional nanoparticles significantly improves electrochemical performance due to the synergistic effects arising from the contributions of individual particles.

### 3.3. Specific Area and Pore Size Distribution

**Figure 4:**
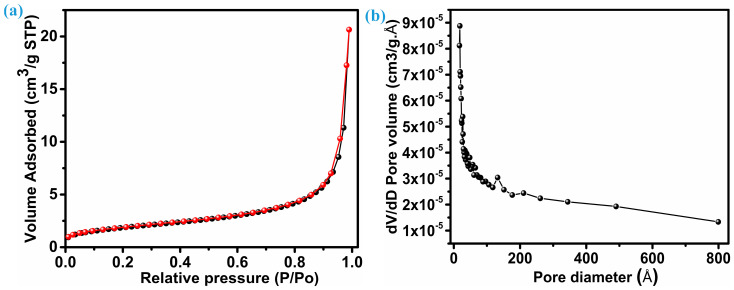
Specific area and pore size distribution, (**a**) BET, and (**b**) pore size distribution.

An electrode’s electrochemical performance is significantly influenced by its surface area. Brunauer–Emmett–Tell (BET) analysis, as shown in Figure 4a, reveals the distribution of specific surface area. On the other hand, Figure 4b reflects the mean pore diameter for the ZnS sample with nanoparticles with multiple dimensions; both figures provide insights into their properties. Following the IUPAC classification, the isotherm pattern (N_2_ adsorption–desorption) of ZnS belongs to the near-type IV isotherm category. This particular type is characteristic of inhomogeneous mesoporous materials that display an H1 hysteresis loop [34,35]. This ZnS sample reflects a comparable surface area of 16.7 m^2^.g^−1^. The considerable surface area demonstrates that ZnS nanoparticles synthesized here have the potential to improve electrolyte permeability, aiding in the efficient transport of ions and electrons within the electrode material. Consequently, this enhances overall electrochemical performance. Furthermore, the mesoporosity of the ZnS sample with hybrid dimension particles was verified by pore size distribution based on the Barrett, Joyner, and Halenda (BJH) curve. As seen in Figure 4b, the average pore diameter for ZnS nanoparticles remains at 217.51 (21.75 nm) Å, and the overall pore volume measures 0.031 cm^3^.g^−1^.

### 3.4. Electrochemical Performance and Charge Transfer Mechanism Analysis

**Figure 5:**
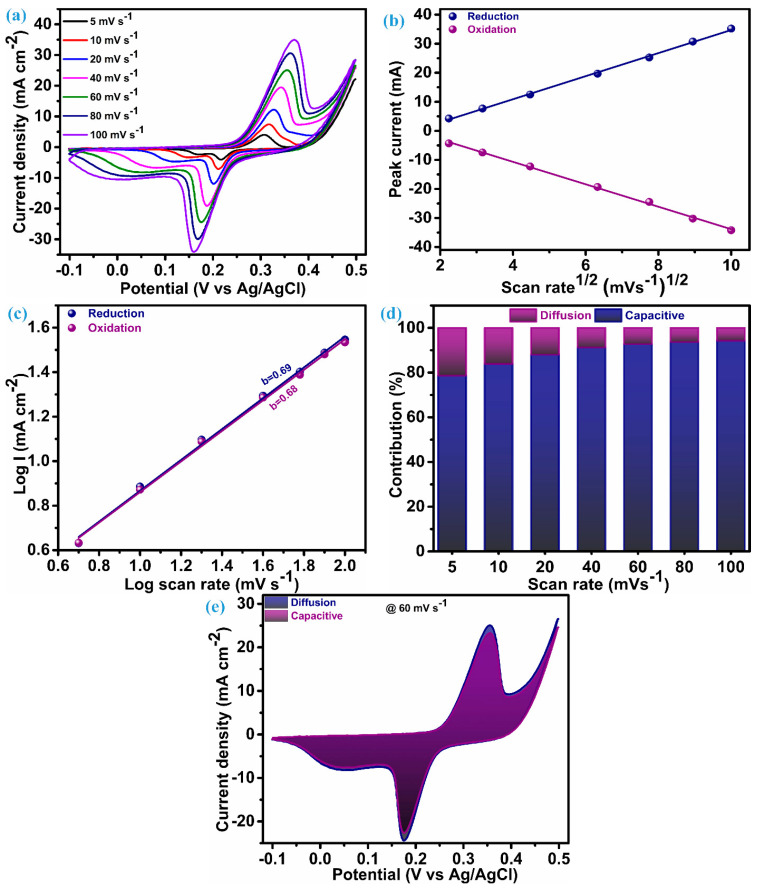
CV and charge transfer analysis, (**a**) CV at different scan rate, (**b**) peak current vs. SQRT of scan rate, (**c**) b-value plot, (**d**) stacked columns of surface and diffusion contribution, and (**e**) CV curve at 60 mV.s^−1^ with area of surface and diffusion.

The electrode fabricated on Ni-foam of ZnS nanoparticles as electroactive material underwent electrochemical testing (CV, GCD, and EIS) within a three-electrode configuration to investigate the charge storage features. Figure 5a presents the cyclic voltammograms acquired for an electrode of ZnS nanoparticles in the potential range of −0.1 to 0.5 V. Cyclic voltammograms are measured by keeping different scan rates between 5 mV.s^−1^ to 100 mV.s^−1^. The cyclic voltammetry (CV) curves at each scan rate present a notable and characteristic pattern featuring a pair of redox peaks. These redox peaks serve as clear indicators, suggesting the hybrid capacitive/battery-type behavior inherent in the material under consideration, i.e., ZnS nanoparticles. This observation underscores the materials’ capacity to store and release electrical energy through reversible redox processes [36,37,38]. The area of the CV curve exhibits a substantial increase as the scan rates escalate, while the shapes of the curve remain consistent, even reaching a higher scan rate. This emphasizes the exceptional electrochemical performance and reduced polarization throughout cycling, attributed to the cluster of ZnS through ZnS nanoparticles [13,18]. In the CV patterns, as the scan rates rise, there are noticeable shifts in the positions of the anodic and cathodic (redox) peaks toward elevated and diminished energy levels, respectively. This rise and fall in potential is accompanied by a concurrent rise in the peak current (i_p_), signifying a swift anion diffusion rate and minimal charge transfer resistance. A based redox process observed through a CV possible electrochemical reaction mechanism for ZnS can be elucidated as [13];

(2) $$ZnS+{OH}^{-}↔ZnSOH+e^{-}$$

(3) $$ZnSOH+{OH}^{-}↔ZnSO+H_{2}O+e^{-}$$

Cyclic voltametric curves play a valuable role in elucidating the impact of the charge storage mechanism linked to electrode materials. To comprehend the overall charge storage mechanism of the material under investigation, it is essential to explore two mechanisms: the surface-controlled process (capacitive process) and the diffusion-controlled or faradaic process. It is perceived that changes in the scan rate usually have little effect on the peak potential when a redox process is purely regulated by a diffusion-controlled mechanism. On the other hand, changes in the scan rates lead to changes in the peak potential during voltammetry if the surface-controlled mechanism is the primary factor of charge storage for that material [39].

To corroborate this conjecture, a comprehensive analysis of the charge transfer kinetics was conducted for this ZnS nanoparticle electrode using its CV profile. The first step of this analysis is initiated with a diffusion coefficient (D) that elucidates penetration of the K^+^ ions in the ZnS during the electrochemical process. This diffusion parameter is derived through the application of the Randles–Sevcik equation as [6];

(4) $$i_{p}=0.4463\times n\times F\times C\times A\times \sqrt{\frac{nFvD}{RT}}$$

Several factors in this equation remain constant, including the Faraday constant (F), molar gas constant (R), and temperature (T) during the measurement. The other parameters are: peak current (*i*_p_), estimated from CV curves; scan rate (*v*), the applied potential; area (A), the electrode area; electrolyte concentration (c); and the number of electrons (n), all of which need to be considered while estimating diffusion parameters. Hence, the aforementioned equation can be expressed as follows;

(5) ipv12=2.69×105×A×C×D12×n12

The value of *_i_*_p_/v12 can be evaluated through the linear fitting of *i_p_* vs. v12 plot, and that is the slope of this plot. The plot of the peak current noted during the reversible redox process vs SQRT of the scan rate for the ZnS sample is illustrated in Figure 5b. For the ZnS sample, the diffusion coefficient (D), estimated through the preceding equation, is 2.72 × 10^−3^ cm^3^ s^−1^ at the reduction peak and 2.68 × 10^−3^ cm^3^ s^−1^ at the oxidation peak. These values of the diffusion coefficient suggest that multidimensional nanoparticle clusters of ZnS act as effective diffusion sites, enhancing the rate of the diffusion process. The linearity of this plot reflects that the charge transfer process of the ZnS sample arises due to the involvement of both mechanisms. Therefore, for identifying the dominating charge transfer kinetics (surface or diffusion controlled) in the ZnS nanoparticles, a power law was employed, as proposed by H. Lindström [40].

(6) $$i\left(v\right)=i_{surface}+i_{diffusion}={av}^{b}$$

*i*(*v*) represents the current at a particular potential for CV curves, measured at various scan rates. Finding the slope between the logarithms of current (log(*i*)) and scan rates (log(*v*)) will yield the value of parameter b, which is known as a constraint of the charge storage mechanism. Two scenarios of the b-value decide the control of the charge governed; specifically, when b equals 1, it is surface-controlled, and when b equals 0.5, it means it is diffusion-controlled. In our case, a log–log plot of current (log(i)) against voltage (log(v)), as shown in Figure 5c, yields a slope of 0.69. This indicates that the charge transfer process exhibits a mixed control type, with the surface-controlled mechanism predominantly influencing the outcome. Consequently, the current arising from the surface and diffusion mechanism in the system can be estimated based on the relation proposed by Liu et al. [41]:

(7) $$i\left(V\right)=k\nu +k^{\prime }\nu^{0.5}$$

The above equation can rearranged as per Dunn et al. [42] and followed for further analysis of the charge storage mechanism as:

(8) iVv12=kν12+k′

Determining values for k and k’ at distinct potentials across various applied scan rates provides a quantitative assessment of the current fraction attributed to the surface- and diffusion-controlled mechanisms, respectively. The stacked column graph, as shown in Figure 5d, represents the current contribution of the ZnS sample at each scan rate as applied for the measurement of CV curves. As seen in Figure 5d, the surface-controlled mechanism dominates, as 78.64% of the total contribution of the current is from this process when the scan rate is at its lowest level, i.e., 5 mV.s^−1^. This contribution of surface current continually increases when the scan rate is elevated further, which is anticipated owing to its more pronounced linear dependence on the scan rate [40]. The cyclic voltammogram of the ZnS sample at a scan rate of 60 mV.s^−1^, outlining the surface-controlled current area (92.73%) and diffusion-controlled current area (7.27%), are represented in Figure 5e.

The performance parameter of the ZnS supercapacitor electrode, i.e., specific capacitance (areal capacitance), was further evaluated using discharge time noted through the charge–discharge profile at various current densities.

(9) $$C_{s}=\frac{I\times t_{d}}{m\times ∆V}$$

(10) $$C_{A}=\frac{I\times t_{d}}{A\times ∆V}$$

**Figure 6:**
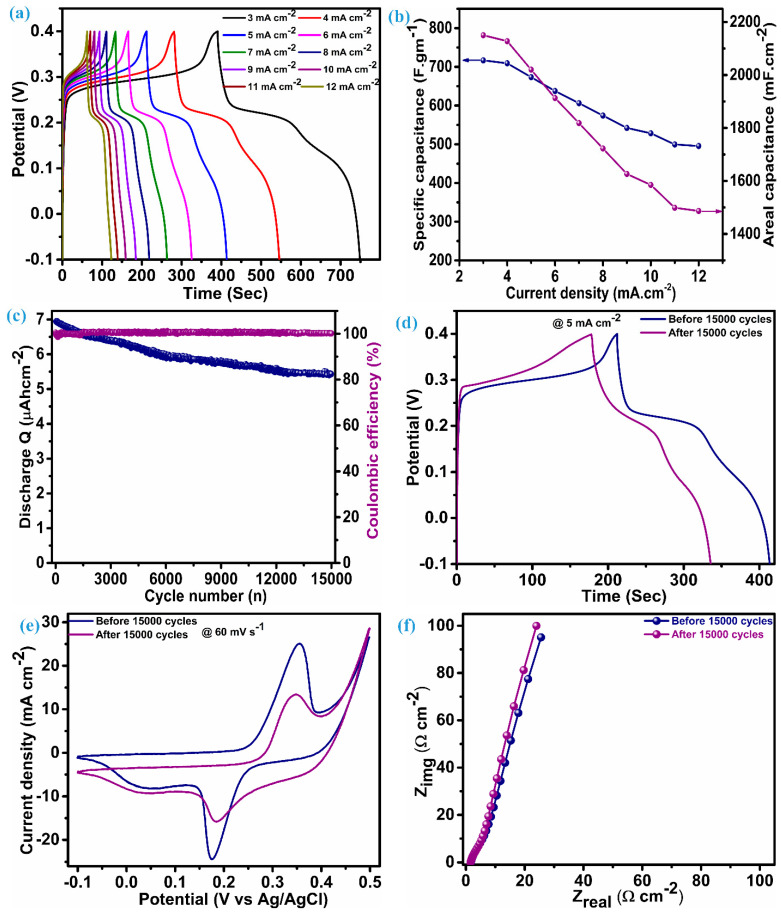
Electrochemical performance analysis, (**a**) charge–discharge profile, (**b**) specific and areal capacitance vs. current density, (**c**) discharge Q and coulombic efficiency, (**d**) charge–discharge at 5 mA.cm^−2^ before and after stability, (**e**) CV at 60 mV.s^−1^ before and after stability, and (**f**) EIS curves before and after stability.

Reckoning C_s_ (specific capacitance) and C_A_ (areal capacitance) is possible by adding the values of discharge time (td), the quantity of active material (m) per unit electrode area (1 cm^2^), and the range of voltage (ΔV). The charge–discharge profile for the ZnS electrode is illustrated in Figure 6a, which has an almost symmetric shape (nearly equal charge-discharge profile), indicating the ability of the electrode material to give larger specific capacitance. An electrode composed of multi-dimensional ZnS nanoparticles demonstrates a specific (areal) capacitance of 716.8 F.g^−1^ (2150.4 mF.cm^−2^) when operating at a current density of 3 mA.cm^−2^. Figure 6b reflects the capacitance (specific and areal) at various current densities for the ZnS electrode; this electrode exhibits notable capacitive features with a specific (areal) capacitance of 495.5 F.g^−1^ (1486.5 mF.cm^−2^), even when the current density was raised fourfold to the initial current density, i.e., 12 mA.cm^−2^. This fascinating feature indicates the material’s ability towards insertion–desertion of electrolytic ions, even at higher current densities. Remarkably, the specific (areal) capacitance values of these electrodes exceed those of ZnS-based electrodes and their composites with other materials. This encompasses WO_3_-ZnS with a capacitance of 215 F.g^−1^ [1], rGO/MWCNTf-ZnS showing a specific capacitance of 95 F.g^−1^ [2], ZnS (501 F.g^−1^) and ZnS-CuSe_2_ (640 F.g^−1^) [15], ZnS microspheres (67.75 F.g^−1^) and Ni-ZnS microspheres (104.2 F.g^−1^) [36], ZnS-Graphene (197.1 F.g^−1^) [43], ZnS microflowers (36.5 mF.cm^−2^), and ZnO-ZnS nanoforests (217 mF.cm^−2^) [44]. Moreover, our electrode, employing multidimensional ZnS nanoparticles, exhibits impressive cycling stability, retaining 77.7% of capacitance, even after enduring 15,000 charge–discharge cycles at a current density of 25 mA·cm⁻², with a coulombic efficiency gradually exceeding 100%. Greater coulombic efficiency conditions indicate reduced internal resistance, shorter ion transport distances, and enhanced utilization of the electroactive surface [45]. This also reflects minimal structural deformation and phase transformation. Figure 6c depicts the stability (discharge Q) and coulombic efficiency over 15,000 cycles for the ZnS electrode. To ensure this, the determination of capacitance was achieved by assessing the charge–discharge curve at a rate of 5 mA.cm^−2^ both before and after 15,000 cycles, as illustrated in Figure 6d. The capacitance achieved after stability at 5 mA.cm^−2^ is 528.6 F.g^−1^ (1585.7 F.cm^−2^), which was 673.4 F.gm^−1^ (2020.2 mF.cm^−2^) before stability. The cyclic voltammetry (CV) was obtained at a scan rate of 60 mV.s^−1^ (Figure 6e), retaining its original shape with minimal loss in the corresponding area, consistent with the findings observed in the galvanostatic charge–discharge (GCD) analysis conducted post stability assessment. Many possible ways need to be considered to achieve improvement in the cycling stability of ZnS. This includes optimized fabrication of the material through varying different parameters, fabrication of binder-free electrodes of ZnS, and fabrication of a ZnS composite with other materials with prolonged cycling stability. The electrochemical impedance spectroscopy (EIS) spectra before and after stability exhibit nearly the same features as illustrated in Figure 6f, which have a linear trend at a low frequency and a semicircular pattern at a high frequency. The series resistance (Rs) for the ZnS electrode is 1.66 Ω.cm^−2^ before stability and 1.58 Ω.cm^−2^. This value can be measured by taking an intercept at the x-axis and suggests excellent electrical conductivity and electroactivity associated with the ZnS electrode. Furthermore, close alignment of a straight line with a y-axis is an indication of predominant capacitive features [14].

## 4. Conclusions

In conclusion, the one-pot solvothermal synthesis of a cluster of ZnS low-dimensional nanoparticles presents a promising avenue for the development of high-performance supercapacitor electrodes. The low-dimensional nature of ZnS clusters offers a high surface area, facilitating rapid charge and discharge kinetics, which are crucial for supercapacitor applications. The successful integration of ZnS nanoparticles into supercapacitor electrodes is underscored by their excellent electrochemical properties, including high specific capacitance, good cyclic stability, and low internal resistance. This research provides a significant understanding of the synthesis and design of novel materials for energy storage applications, opening the door to the creation of high-performance supercapacitors with increased longevity and efficiency.

## Data Availability

Data are contained within the article.
